# Modeling Tumor-Host Interactions of Chronic Lymphocytic Leukemia in Xenografted Mice to Study Tumor Biology and Evaluate Targeted Therapy

**DOI:** 10.1038/leu.2013.131

**Published:** 2013-04-26

**Authors:** Sarah E. M. Herman, Xiameng Sun, Erin M. McAuley, Matthew M. Hsieh, Stefania Pittaluga, Mark Raffeld, Delong Liu, Keyvan Keyvanfar, Colby M. Chapman, Jichun Chen, Joseph J. Buggy, Georg Aue, John F. Tisdale, Patricia Pérez-Galán, Adrian Wiestner

**Affiliations:** 1 Hematology Branch, National Heart, Lung, and Blood Institute, National Institutes of Health, Bethesda, MD; 2 Laboratory of Pathology, National Cancer Institute, National Institutes of Health, Bethesda, MD; 3 Pharmacyclics Inc., Sunnyvale, CA, USA; 4 Department of Hemato-Oncology. Institut d'Investigacions Biomédiques August Pi I Sunyer (IDIBAPS), Barcelona, Spain

**Keywords:** Ibrutinib, BCR, CLL, Xenograft, NF-kappaB, Kinase Inhibitor

## Abstract

Chronic lymphocytic leukemia (CLL) cells depend on microenvironmental factors for proliferation and survival. In particular, the B-cell receptor (BCR) and NF-κB pathways are activated in the lymph node microenvironment. Thus, model systems mimicking tumor-host interactions are important tools to study CLL biology and pathogenesis. We investigated whether the recently established NOD/scid/γc^null^ (NSG) mouse xenograft model can recapitulate the effects of the human microenvironment. We assessed, therefore, tumor characteristics previously defined in lymph node-resident CLL cells, including proliferation, and activation of the BCR and NF-κB pathways. We found that the murine spleen microenvironment supported CLL cell proliferation and activation to a similar degree than the human lymph node, including induction of BCR and NF-κB signaling in the xenografted cells. Next, we used this model to study ibrutinib, a Bruton's tyrosine kinase inhibitor in clinical development. Ibrutinib inhibited BCR and NF-κB signaling induced by the microenvironment, decreased proliferation, induced apoptosis, and reduced the tumor burden *in vivo*. Thus, our data demonstrate that the spleen of xenografted NSG mice can, in part, recapitulate the role of the human lymph node for CLL cells. In addition, we show that ibrutinib effectively disrupts tumor-host interactions essential for CLL cell proliferation and survival *in vivo*.

## Introduction

Chronic lymphocytic leukemia (CLL), is characterized by the progressive accumulation of nonfunctional mature B-cells in blood, bone marrow (BM), and lymphoid tissues.^1^ The majority of the tumor cells in the blood are resting; however, *in vivo* measurements demonstrated that up to 1% of the clonal cells are newly generated each day.^2^ This tumor proliferation occurs primarily in tissue compartments such as the lymph node (LN) and BM,^3-6^ often in anatomic structures referred to as “proliferation centers”, where tumor cells co-localize with other cells, in particular T-cells and stromal cells.^1^ In contrast to circulating CLL cells, tumor cells in LN and BM show phenotypic characteristics of activated B-cells and express gene signatures indicating activation of the B-cell receptor (BCR) and NF-κB pathway.^3^ Thus, the biology of CLL cells *in vivo* depends on their anatomic location and is shaped by interactions with components of the tissue-microenvironment. The dependence of CLL cells on tumor-host interactions is underscored by the fact that CLL cells *in vitro* rapidly undergo apoptosis unless substitute microenvironmental factors are provided.^1, 5, 7^

*In vitro*, different types of stromal cells and monocyte derived cells, designated “nurse-like-cells” (NLC), promote CLL cell survival.^5, 7, 8^ Many factors have been found to enhance CLL cell survival and promote limited proliferation *in vitro* including BCR activation, Toll-like receptors (TLR), cytokines, chemokines, CD40L, BAFF, integrins and components of the extracellular matrix.^8-14^ Among these the BCR is increasingly emerging as the pivotal pathway.^15, 16^ A role for BCR signaling in the pathogenesis of CLL has been suggested by observations that CLL cells use a restricted repertoire of *IGHV* genes.^17, 18^ Furthermore, some cases express virtually identical BCRs, so called “stereotyped BCRs”, that recognize shared antigens.^19, 20^ In many cases these may be autoantigens expressed by dying cells.^21^ Comparing purified CLL cells isolated concomitantly from the peripheral blood (PB), BM, and LN of patients we recently showed that CLL cells in the LN contain increased levels of activated SYK and express genes upregulated in response to BCR activation. This indicates that antigenic signaling continues throughout the disease course and that the BCR is engaged primarily in the LN, rather than in the PB.^3^ Consistent with chronic antigen contact *in vivo* is the observation of a reversible down-modulation of surface IgM expression on CLL cells and the resemblance of these cells to anergic B-cells.^22, 23^

Inhibitors of kinases involved in BCR signal transduction have demonstrated substantial clinical activity.^15, 16, 24, 25^ Fostamatinib, a SYK inhibitor, induced objective responses in 55% of CLL patients within two months of starting treatment.^25^ Even higher response rates have been reported for GS-1101 (formerly CAL-101), an inhibitor of PI3Kδ, and for ibrutinib, an irreversible inhibitor of BTK.^24, 26-28^ Ibrutinib induced objective responses in 60% of patients with relapsed B-cell malignancies.^24^ Interestingly, CLL patients had the highest response rate at 79%, and responses appear to be quite durable.^24, 29^ As first noted with fostamatinib,^25^ BCR directed therapies result in an initial, transient increase in the absolute lymphocyte count in the PB.^26-28, 30^ *In vitro*, ibrutinib inhibits BCR signaling, induces apoptosis, prevents proliferation of activated CLL cells, and inhibits migration of CLL cells to chemokines and adhesion to stromal components.^31-34^ *Ex vivo* analysis of CLL cells from the PB of patients treated with fostamatinib demonstrated inhibition of BCR signaling and reduced tumor proliferation, with no apparent correlation between the degree of inhibition and clinical response, suggesting that analysis of tissue samples will be important to assess activity of BCR targeted agents *in vivo*.^35^

Modeling tumor-host interactions is an area of intense investigation. Such models are of particular interest given the fact that tissue resident CLL cells are not readily available. Currently, the most widely utilized *in vivo* model for CLL is the transgenic TCL1 mouse, in which the human *TCL1* gene is expressed under the control of the immunoglobulin heavy chain variable region promoter and enhancer.^36^ More recently, a knockout mouse model recapitulating the chromosomal deletion at 13q14 has been established.^37^ While both of these mouse strains model CLL, they are based on either the overexpression of an oncogene or the deletion of a specific regulatory region and as such represent a specific disease genotype. A complementary approach has been to xenograft the Mec-1 cell line^38^ or primary CLL cells into immune-compromised mice.^39, 40^ Recently, Bagnara et al. reported that peripheral blood mononuclear cells (PBMCs) from CLL patients xenografted into NOD/scid/γc ^null^ (NSG) mice localized primarily to the murine spleen. Furthermore, these investigators found that proliferation of CLL cells *in vivo* was dependent on co-engrafted human T-cells. Similarly, using a NOD/scid CLL xenograft model, Aydin et al. demonstrated tumor engraftment in the murine spleen.^41^ Thus, these xenograft models may be suitable to study tumor microenvironment interactions.

In order to determine whether xenografted CLL cells in NSG mice share the biologic characteristics of CLL cells in the human LN microenvironment we compared CLL cells isolated from spleens of xenografted NSG mice to CLL cells from human blood and LN. In CLL cells isolated from the human and murine hosts we measured activation of BCR and NF-κB pathways, tumor proliferation, and the expression of immunophenotypic markers of cellular activation. After having confirmed that this NSG xenograft model recapitulates key elements of CLL biology, we used this system to study the impact of the BTK inhibitor ibrutinib on tumor biology *in vivo*.

## Materials and Methods

### Patient samples, xenotransplantation of NSG mice, and ibrutinib treatment

Matched PBMCs and LN biopsies were obtained from previously untreated CLL patients (Table 1) in accordance with the Declaration of Helsinki.^3^ For xenografting experiments we choose samples with sufficient viably frozen cells and, where possible, samples from patients who had previously contributed lymph node biopsies for gene expression studies. PBMCs were prepared by density-gradient centrifugation (Ficoll Lymphocyte Separation Media; ICN Biomedicals, Irvine, CA, USA) and viably frozen in 90% fetal bovine serum (FBS), 10% dimethyl sulfoxide (DSMO) (Sigma, St. Louis, MA, USA) in liquid nitrogen. Xenografting of PBMCs into NSG mice (Jackson Laboratory, Bar Harbor, ME, USA) was carried out as described by Bagnara et al.^39^ with modifications: we did not adoptively transfer any hematopoietic cells other than the CLL patient's PBMCs, and 25 mg/kg of busulfan (Busulfex, Otsuka America Pharmaceuticals, Inc., Rockville, MD, USA) given intraperitoneally (i.p.) on day -1 was used in lieu of irradiation to condition the mice 24 hours before intravenous (i.v.) injection of 1x10^8^ PBMCs. PBMCs were thawed in RPMI (Gibco, Grand Island, NY, USA) with 10% FBS (hereafter R10) and in some experiments stained with 0.5 μM CFSE (Carboxyfluorescein succinimidyl ester, Invitrogen, Grand Island, NY, USA) as described.^42^ Viably frozen cells used in all experiments had an initial viability of at least 80%; with B-cells comprising 85-97% and T-cells comprising 1-15% of the PBMC sample. In each experiment, 2-5 mice (per treatment group) were injected with cells from the same patient. For most patients at least two independent sets of experiments were conducted. Ibrutinib (provided by Pharmacyclics, Inc., Sunnyvale, CA, USA) was added to the drinking water at 0.16 mg/ml starting the day before PBMC injection (day -1) until sacrifice 3-4 weeks post xenograft. As described this regimen results in an average ibrutinib dose of 25mg/kg/day, which is sufficient to achieve 90% occupancy of BTK and clinical efficacy in mouse models.^33, 43^ Controls were given water containing vehicle alone (1% HP-beta-cyclodextrin).

### Collection and processing of cells from mice

#### Mice were bled weekly or biweekly until sacrifice

In all experiments except for data displayed in Supplemental Figure S1 sacrifice was 3-4 weeks post xenografting. BM cells were harvested by flushing the femoral and tibial bones with R10 media and splenocytes were obtained by homogenizing harvested spleens; these samples were then filtered through 70 μm nylon sieves (BD Falcon, Franklin Lakes, NJ, USA). Erythrocytes were lysed using ACK buffer (Quality Biological, Inc., Gaithersburg, MD, USA).

### Immunohistochemistry

Spleen tissue pieces were fixed in 10% formalin, paraffin embedded, sectioned, and stained with hematoxylin and eosin (H&E) or with human anti- CD3, CD5, CD20 or MIB (Ki67) antibodies. Images were collected using the Olympus Bx41 microscope (Center Valley, PA, USA) at the indicated magnifications.

### Flow cytometry

PBMCs and tissue derived single cell suspensions were stained using either the IntraSure™ kit or the Phosflow Fix/Perm buffer I/III kit (for phosphorylation analysis) (BD Biosciences, Franklin Lakes, NJ, USA). Briefly, up to 0.5 × 10^6^ cells were washed, resuspended in FACS buffer (DPBS with 5% FBS) and incubated (30 minutes at 4°C) with the appropriate human antibodies: CD45-APC, CD19-PECy5, CD5-PECy7 and one of the following: IgG1-PE, CD38-PE, CD69-PE, or CD184-PE (BD Biosciences). CLL cells were identified as CD45^+^/CD19^+^/CD5^+^, non-CLL B-cells as CD45^+^/CD19^+^/CD5^-^, and T-cells as CD45^+^/CD19^-^/CD5^+^. Post surface staining, either Ki67 staining was done using the IntraSure™ Kit, or the cells were fixed, permeabilized and stained with one of the following Alexa488 conjugated antibodies: IgG1, pBTK(Y551), pPLCγ2(Y759) or pERK(T202/Y204) (BD Biosciences). To determine cell viability, cells were incubated with LIVE/DEAD fixable violet solution (Invitrogen). Cells were analyzed on a FACS Canto II flow cytometer (BD Biosciences) using FACS-DIVA 6.1.1 and FlowJo software (Version 8.8.6; TreeStar, Ashland, OR, USA). Determination of absolute cell counts per μL PB was done by adding AccuCount blank particles (Spherotech, Lake Forest, IL, USA). The abundance of human cells in the BM and spleen was measured as the percentage of human cells (defined by CD45 staining) among all nucleated cells (defined by forward and side scatter properties) as previously described.^39^

### Gene expression

CLL cells from the PB, human LN, and murine spleen were purified by CD19^+^ selection using MACS Cell Separation Columns (Miltenyi Biotec, Cambridge, MA, USA). Total RNA of purified tumor cells (>96% pure CLL) was extracted using RNeasy kits (QIAGEN, Valencia, CA, USA) and cDNA was prepared using the High Capacity cDNA RT Kit (Applied Biosystems, Carlsbad, CA, USA). Quantitative RT-PCR was run using TaqMan Custom Arrays on microfluidic cards on an ABI PRISM 7900HT Sequence Detection System (Applied Biosystems; Supplementary Table S1). Relative expression reflects the ΔΔCt values, where ΔCt is defined as the Ct of the housekeeping gene (*VCP*) – Ct of the gene of interest (for example *EGR1*) and ΔΔCt is defined by the ΔCt in the patient's LN (or mouse SP) minus the ΔCt in the patient's PB.

### Statistical analysis

To compare non-random measurements in an individual mouse either across time or compartment, a paired Student's t-test was used (Prism5, GraphPad, La Jolla, CA, USA). Groups of mice grafted with cells from one patient were averaged when comparing parameters related to the patient and a paired Student's t-test was applied. In cases with multiple variables the linear model y_ijk_ = μ + treatment_i_ + subject_j_ + e_ijk_ was used to fit the data, with y_ijk_ being the observation in the ith treatment, the jth subject, and the kth replicate, μ the general mean and e_ijk_ is the residual error (JMP9, SAS Institute, Cary, NC, USA).

## Results

### Xenografted human CLL cells preferentially reside and proliferate in the spleen of NSG mice

We injected 1x10^8^ human PBMCs from CLL patients into NSG mice and obtained repeat blood counts. After one week the CLL count in the PB of the NSG mice averaged 297 cells/μl and the T-cell count averaged 41 cells/μl. Within the next 2-3 weeks the PB CLL cell count decreased, while the PB T-cell count remained constant (Supplementary Figure S1a). Three to four weeks after xenografting CLL cells localized predominantly to the murine spleen contributing on average 7% of the total cellular elements and >50% of the human cells in this compartment. In contrast, CLL cells contributed <0.5% of the total cellular elements in the BM and only 30% of the human cells (Supplementary Figure S1b and data not shown). Immunohistochemistry (IHC) showed CLL infiltration of the murine spleen in a nodular pattern, typically surrounding blood vessels (Supplementary Figure S1c). A rim of CD3+ T-cells was often seen around the CLL aggregate, with some T-cells intermixed with the tumor. Ki67 stained proliferating cells were predominantly localized in the lymphoid aggregates (Supplementary Figure S1d).

**Figure 1:**
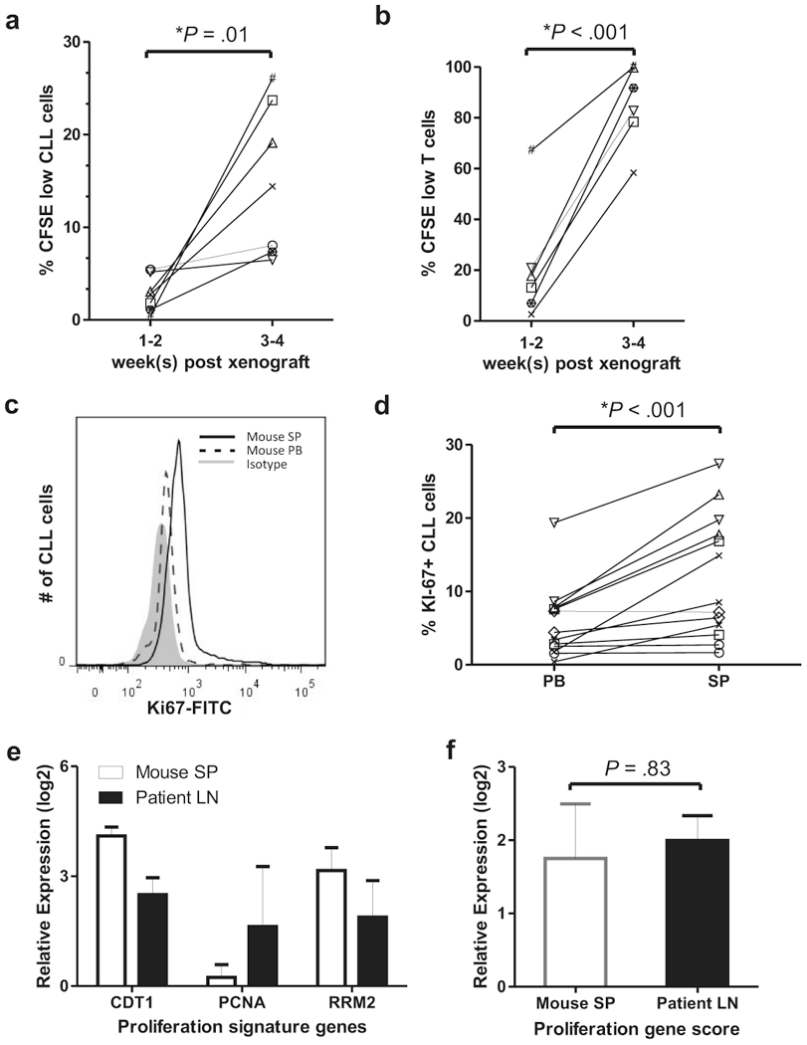
Xenografted human CLL cells proliferate in the spleen of NSG mice. (**a, b**) The fraction of human cells having undergone cell division increases with time from xenografting. PBMCs from seven CLL patients (represented by a unique symbol; Table 1) were labeled with 0.5 μM CFSE before injection into NSG mice. The percentage of human CLL (CD45^+^, CD19^+^, CD5^+^; in **a**) or T-cells (CD45^+^, CD19^−^, CD5^+^; in **b**) having undergone cell division (low CFSE staining) is shown in peripheral blood (PB) samples at 2-week intervals (2-5 mice per patient). (**c-e**) Mice were sacrificed 3-4 weeks post xenografting. (**c**) A representative histogram demonstrates increased Ki67 staining in CLL cells from the spleen (SP) compared to cells in the PB. (**d**) The percentage of Ki67 positive CLL cells is higher in the spleen than in the PB. Lines connect PB and spleen samples from the same mouse (n = 13; symbols identify individual patients; Table 1). (**e**) CLL cells in secondary lymphoid tissues upregulate genes typically expressed in proliferating cells. Shown is the mean (± SEM) expression for each gene in CLL cells from the indicated tissue normalized to its expression in the corresponding PB cells. CLL cells were CD19+ purified from the spleen of xenografted mice (2-4 spleens per patient) and from the corresponding patients' LN and PB (n=3).^3^ (f) The mean (± SEM) proliferation gene score, computed as the averaged expression of *CDT1, PCNA, and RRM2* shown in (e), is comparable between CLL cells from the mouse spleen and the human LN. Student's paired t-test was used to test for significance in all panels.

In order to measure CLL and T-cell proliferation we injected CFSE stained PBMCs and determined the fraction of proliferating cells identified by a decrease in CFSE staining on flow cytometry (Supplementary Figure S2a-b). One to two weeks after xenografting <5% of the circulating CLL cells showed decreased CFSE staining. However this proliferating fraction increased significantly by weeks 3-4 (Figure 1a; *P*=.01), consistent with a delayed onset of tumor proliferation as described previously.^39^ Proliferation of T-cells was also delayed but once established, was faster than in the CLL cells (Figure 1b). The growth rate of CLL cells ranged from 0.23% to 0.91% of the clonal cells per day (estimated from the fraction of cells with low CFSE staining divided by the number of days post xenografting). This range is in good agreement with the proliferation rate found in patients using deuterium labeling.^2^ The four tumor samples with the highest growth rates were IGHV unmutated, whereas two of three samples with relatively lower proliferation were IGHV mutated (Figure 1a, Table 1). We did not find a correlation between T-cell and CLL cell proliferation rates; however as previously reported,^39^ CLL proliferation appeared to depend on the presence of autologous T-cells (data not shown). Thus, despite simplifications in the xenografting protocol, the tissue localization and proliferation kinetics of the xenografted CLL cells are in agreement with findings by Bagnara et al.^39^

We then sought to compare the proliferation rate of xenografted cells in blood and spleen. The fraction of CFSE low CLL cells was only slightly increased in the spleen compared to the PB (data not shown). As the CFSE method identifies cells that have undergone cell division, not cells that are in cell cycle, trafficking of new born cells between different sites may obscure any tissue specific differences in proliferation rate. We therefore used Ki67 staining by flow cytometry to estimate the proportion of actively cycling cells in each compartment. As shown in a representative histogram in Figure 1c, the percentage of Ki67 positive CLL cells was higher in the mouse spleen than in the blood. In summary, the spleen contained a significantly higher fraction of actively cycling CLL cells than the PB (Figure 1d, P<.001). Interestingly, T-cells also demonstrated increased proliferation in the mouse spleen compared to the PB (Supplementary Figure S3a-b, *P*<.001).

In order to directly compare the proliferation rate of CLL cells in the murine spleen to that in the corresponding patient's LN, we analyzed xenografted CLL cells from three patients that have previously donated PB and LN.^3^ In purified CLL cells we measured expression of three genes that are upregulated in proliferating cells and that we have previously shown to be expressed in the human LN.^3^ The mRNA level for each gene (CDT1, PCNA, and RRM2) was determined by quantitative RT-PCR (Table S1). For each patient three distinct sample populations were analyzed: xenografted CLL cells harvested from the mouse spleen, CLL cells isolated from the human LN, and CLL cells from the PB sampled at the time of the LN biopsy. For each gene the mRNA level in CLL cells from a tissue site was normalized to the mRNA level in the matched PB sample resulting in a relative expression score for each gene (Figure 1e). By averaging the expression score of each of the genes, we derived a proliferation gene score as a quantitative measure of tumor proliferation.^44^ The proliferation score of CLL cells in secondary lymphoid tissues was 3-4-fold higher than in the PB, and was similar in human LN and mouse spleen (Figure 1f), indicating that the splenic microenvironment of xenografted mice is sufficient to support CLL proliferation at a similar rate as the human microenvironment.

### Xenografted CLL cells in the mouse spleen show immunophenotypic changes of activated cells

**Figure 2:**
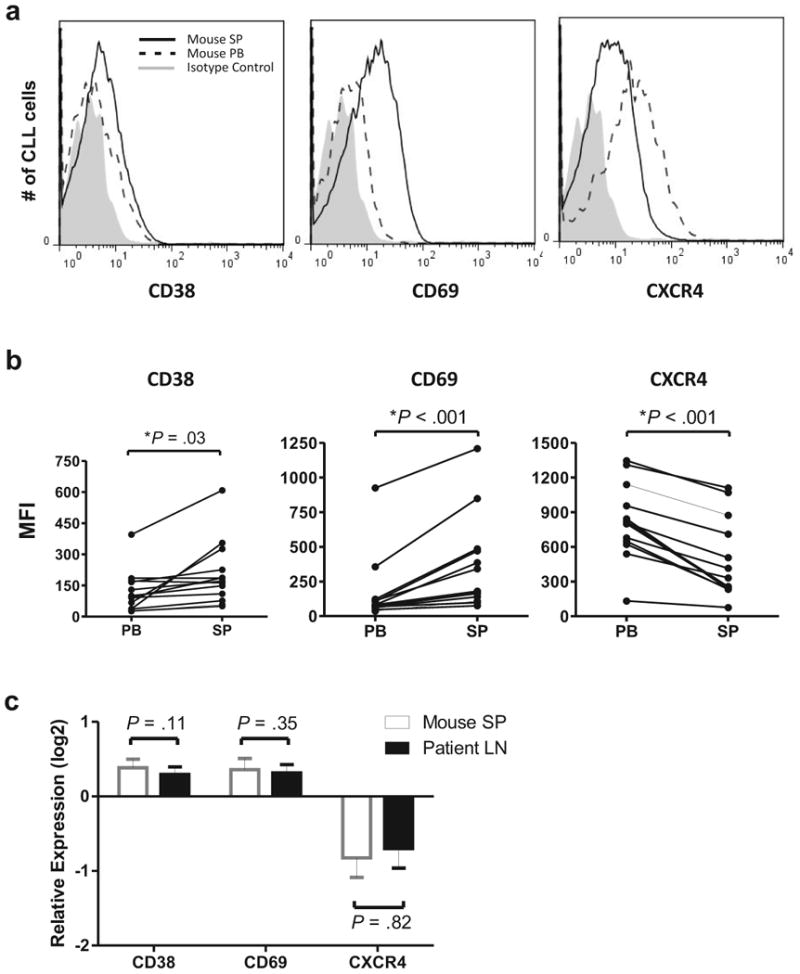
Expression of activation markers on CLL cells is increased in the tissue compartment. (**a**) Overlay histograms showing mean fluorescent intensity (MFI) for CD38, CD69, and CXCR4 (CD184) on xenografted CLL cells from the mouse spleen (SP) and PB. (**b**) NSG mice (n=13) injected with PBMCs from six different patients were sacrificed 3–4 weeks post xenografting. Each dot represents a sample from one mouse; lines connect PB and SP samples from the same mouse. (**c**) CLL cells in the mouse spleen (SP) and human lymph node (LN) upregulate activation markers and downregulate CXCR4 compared to circulating cells in the PB. Shown is the mean (± SEM) of the relative expression for the indicated cell surface markers calculated as the MFI ratio of xenografted CLL cells in mouse SP to mouse PB (open bars) and of CLL cells in human LN to human PB (black bars) obtained from 3 patients. Student's t-test was used to test for significance in all panels.

In keeping with observations in patients, we found that xenografted CLL cells in the murine spleen as compared to matched PB samples showed significantly increased expression of the activation markers CD38 and CD69 (Figure 2a-b, *P* = .03 and *P* < .001, respectively). Similarly, CLL cells in the murine spleen expressed less CXCR4 than circulating CLL cells (Figure 2a-b, *P* < .001), likely as a result of binding to its ligand CXCL12/SDF-1.

To directly compare the effect of the tissue microenvironment on CLL cell activation in mice and man, we assessed the changes in CD38, CD69, and CXCR4 expression between blood and tissue in matched samples donated by the same patient (murine spleen to murine PB; human LN to human PB). As shown in Figure 2c, the changes in all three markers were comparable between xenografted CLL cells in mice and the corresponding patients' *ex vivo* samples.

### Xenografted CLL cells are activated and signal through the BCR and NF-κB pathways in the murine spleen

**Figure 3:**
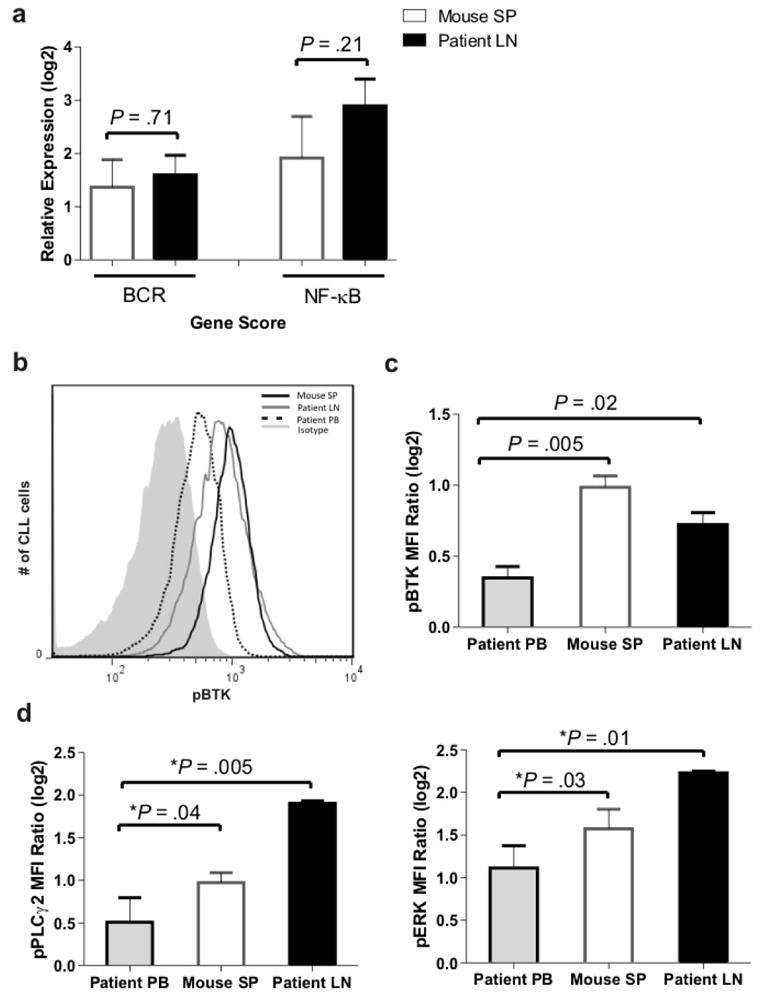
The BCR and NF-κB pathways are activated in xenografted CLL cells in the murine spleen. (**a**) BCR and NF-κB target genes are upregulated in xenografted CLL cells from the murine spleen and in CLL cells isolated from human LN. Shown is the mean (± SEM) of the expression average of representative target genes (described in ^3^, listed in Supplementary Table S1) in CLL cells in tissue sites normalized to that in the PB of the corresponding patients as described in the Materials and Methods. CLL cells from the spleens of xenografted mice (2-4 spleens per patient) and from the LN and PB of the same patients (n=3) were CD19+ purified. Student's paired t-test was used to test for significance. (**b**) Overlay histogram showing increased phosphorylation of BTK (pBTK) in CLL cells from both the mouse spleen (SP) and human lymph node (LN) compared to the matched patient's PB. (**c**) Mean (± SEM) of the MFI ratio of pBTK to isotype control in human PB CLL cells and matched human LN and xenografted CLL cells from mouse spleen (SP) (n=6). (**d**) Mean (± SEM) of the MFI ratio of pPCLγ2 (left panel) and pERK (right panel) to isotype control as in panel c. The multivariable analysis used to test for significance in panels c and d is described in Materials and Methods.

LN-resident CLL cells show activation of the BCR and NF-κB pathways resulting in the upregulation of characteristic gene signatures.^3^ Whether these pathways are also activated in xenografted CLL cells in NSG mice has not been determined. We selected 13 genes representative of gene signatures regulated by BCR and NF-κB activation (Supplementary Table S1) and measured their expression by quantitative PCR. Eleven of these 13 genes were upregulated in xenografted CLL cells from the murine spleen compared to circulating CLL cells in the patient's PB (Supplementary Figure S4a-b). As a quantitative measure of pathway activation, the mRNA expression levels of the respective target genes were averaged into a BCR and NF-κB gene score. Remarkably, the average increase in gene expression of BCR and NF-κB target genes in mice approximated that in the human LN (Figure 3a).

Next we measured the activation of BCR signaling components using flow cytometry. We first evaluated the phosphorylation of BTK a proximal event upon BCR engagement. A representative histogram demonstrating increased phosphorylation of BTK (pBTK-Y551) in CLL cells from both the mouse spleen and the human LN compared to the matched human PB samples is shown in Figure 3b. CLL cells in the murine spleen and the patient LN expressed significantly more activated BTK (Figure 3c; *P*=.005 and *P*=.02, respectively) than the corresponding CLL cells from the human PB used for xenografting. To corroborate this finding we also evaluated the phosphorylation of PLCγ2, a direct target of BTK, and ERK, a kinase in the MAPK pathway activated upon BCR engagement. Phosphorylation of both PLCγ2 (pPLCγ2-Y759) and ERK (pERK-T202/Y204) were significantly increased in CLL cells in both the murine SP and the human LN compared to cells from the PB (Figure 3d). Together these data indicate that the mouse spleen can provide a microenvironment conducive to CLL cell activation and induction of the BCR and NF-κB pathways.

### Ibrutinib reduces tumor burden and CLL cell viability in the NSG xenografted mice

**Figure 4:**
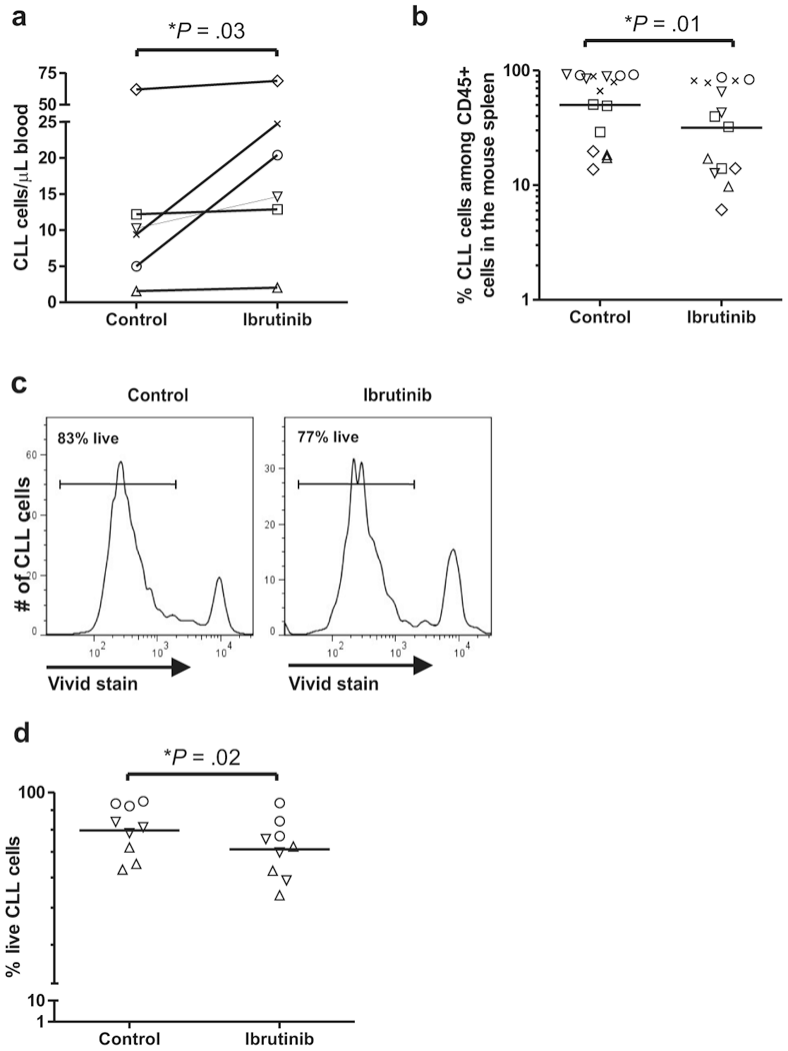
Ibrutinib reduces tumor burden and CLL cell viability in the NSG mice. NSG mice (n=40) injected with PBMCs from six patients were treated with vehicle (control) or ibrutinib and sacrificed 3-4 weeks later. (**a**) The absolute human CLL cell count in the PB of ibrutinib treated mice is higher than in control mice. Data points represent the average measurements of 2-5 mice injected with PBMC from the same patient, identified by unique symbols (Table 1). (**b**) CLL cell infiltration in the murine spleen is reduced by ibrutinib. Each data point represents one mouse; symbols identify individual patients (Table 1). (**c**) A representative histogram demonstrates decreased viability (measured by VIVID Live/Dead stain) in CLL cells in the spleen of an ibrutinib treated mouse (right panel) compared to a control mouse (left panel). (**d**) Decreased viability of CLL cells in the spleen of ibrutinib treated mice. Each data point represents one mouse; symbols identify patients (Table 1).

While Bagnara et al showed that depletion of T-cells in the xenografted NSG mice inhibits CLL cell proliferation,^39^ the effect of the emerging BCR directed therapies have not been investigated in this model. We therefore treated NSG mice with the BTK inhibitor ibrutinib to determine its *in vivo* effects on xenografted CLL cells. In keeping with observations that many patients show a transient increase in the absolute lymphocyte count at the start of ibrutinib therapy,^26, 28^ the CLL cell count in the PB of treated mice was higher than in untreated mice (Figure 4a). Concurrent with this increase of CLL cells in the PB, there was a statistically significant decrease in tumor cells in the spleens of ibrutinib treated mice (average reduction 23%, Figure 4b; *P* = .01). No significant change in T-cell numbers was observed in any of the mice (data not shown). This was further demonstrated by IHC as shown in Supplementary Figure S5.

To determine whether ibrutinib induced cell death *in vivo* we measured tumor cell viability in the mouse spleen using the Vivid dye exclusion assay. Figure 4c shows a representative histogram demonstrating the decrease in cell viability in an ibrutinib treated mouse compared to a control mouse. Overall, we observed a small, but statistically significant reduction in the viability of CLL cells with ibrutinib treatment (average reduction 12%, Figure 4d; *P*=.02). This reduction in viable cells, while small, is consistent with the moderate degree of apoptosis observed *in vitro*.^31, 33^

### Ibrutinib inhibits BCR and NF-κB activation in xenografted CLL cells

**Figure 5:**
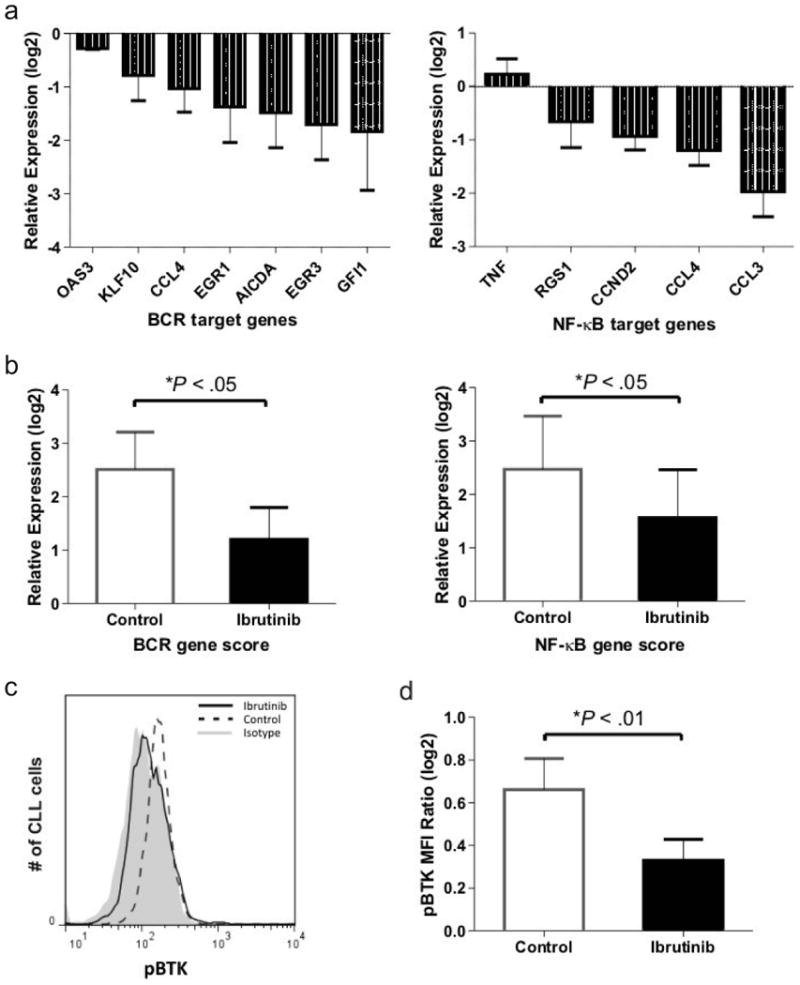
Ibrutinib inhibits BCR and NF-κB signaling in xenografted CLL cells. Mice treated with vehicle (control) or ibrutinib were sacrificed 3-4 weeks post xenografting. (**a**) CLL cells of ibrutinib treated mice as compared to untreated control mice (n=6 per treatment group) show decreased expression of representative BCR (left panel) and NF-κB (right panel) target genes (described in ^3^). Shown is the mean (± SEM) ratio between mRNA levels for each of the indicated genes in CLL cells purified from the spleen of treated as compared to untreated mice. (**b**) The signature scores for the BCR and NF-κB pathways were computed as the averaged expression of the respective target genes (as in Figure 3a). Shown is the relative expression in CLL cells from the spleen normalized to PB. (**c**) A representative histogram shows a decrease of BTK phosphorylation (pBTK) in CLL cells of treated mice. Note: pBTK in treated mice is reduced to the level of isotype control. (**d**) Shown is the mean (± SEM) MFI ratio of pBTK to isotype control in CLL cells from control and ibrutinib treated mice. The multivariable analysis used to test for significance in all panels is described in Materials and Methods.

Next, we sought to determine whether ibrutinib prevents activation of the BCR and NF-κB pathways in CLL cells *in vivo*. We analyzed tumor cells isolated from murine spleens 3-4 weeks after xenografting and measured expression of representative BCR and NF-κB target genes. Expression of all BCR and of four out of five NF-κB target genes were reduced in CLL cells of the ibrutinib treated mice as compared to control mice (Figure 5a), resulting in a decrease of the BCR and NF-κB gene scores by 61% and 47%, respectively (Figure 5b; *P*<.05).

To confirm inhibition of BTK we measured pBTK in CLL cells from the murine spleen by flow cytometry. A representative histogram is shown in Figure 5c. Ibrutinib substantially decreased BTK activation in treated compared to control mice (Figure 5d; *P*<.01). Taken together these data show that ibrutinib effectively inhibits BCR and NF-κB activation in CLL cells in the tissue microenvironment.

### Ibrutinib inhibits CLL proliferation in vivo

**Figure 6:**
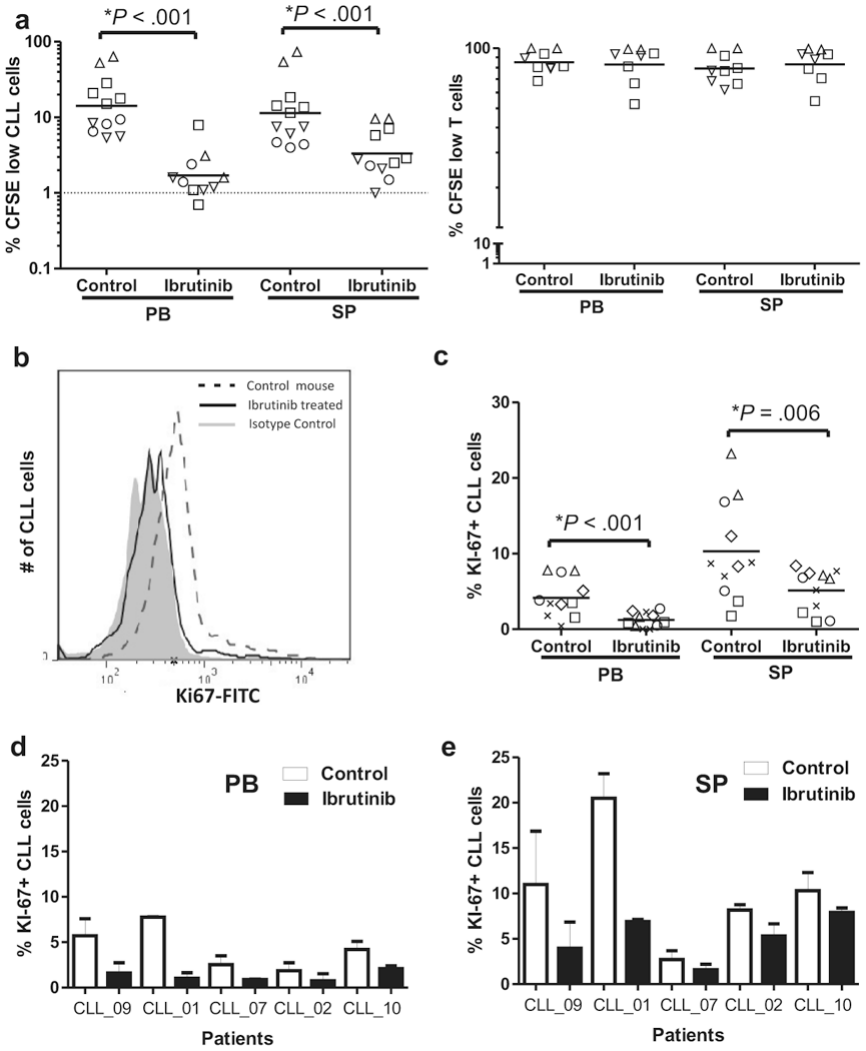
Ibrutinib inhibits CLL but not T-cell proliferation in vivo. (**a**) NSG mice (n=24) injected with CFSE labeled PBMCs from six patients were treated with vehicle (control) or ibrutinib and sacrificed 3-4 weeks later. Symbols identify patients (Table 1), and each data point represents one mouse. The percentage of CFSE low cells (cells having completed cell division) in PB and spleen (SP) for each treatment group is shown for CLL cells (left panel) and T-cells (right panel). In one patient (coded by open circles) the T-cell count in the PB was too low for analysis. (**b**) Overlay histogram showing decreased expression of Ki67 in CLL cells from the mouse spleen of treated as compared to untreated mice at time of sacrifice, 3-4 weeks post xenograft. (**c**) NSG mice (n=22) injected with unlabeled (no CFSE) PBMCs from five patients were treated with vehicle or ibrutinib and sacrificed 3-4 weeks later. Ibrutinib significantly decreased the percentage of Ki67+ CLL cells in PB and spleen (SP). The multivariable analysis used to test for significance in all panels is described in Materials and Methods. (d) The percentage of Ki67+ CLL cells is decreased by ibrutinib in all patients studied. Shown is the mean (± SEM) percentage Ki67+ CLL cells in PB (left panel) and spleen (right panel) of 2-3 mice in each treatment group injected with cells from the five different patients.

Finally, we determined the effect of ibrutinib on tumor proliferation. Ibrutinib decreased CLL cell proliferation measured using CFSE dilution by >80% compared to controls (Figure 6a and Supplementary Figure S6a; *P*<.001). In contrast, ibrutinib had no effect on T-cell proliferation (Figure 6a and Supplementary Figure S6b). In order to assess the proportion of actively cycling cells we measured Ki67 by flow cytometry and determined the percentage of CLL cells that expressed Ki67 (Supplementary Figure S7). Figure 6b shows a representative histogram demonstrating the reduction in Ki67 in CLL cells from ibrutinib treated mice compared to control. Ibrutinib significantly inhibited tumor proliferation as reflected in decreased Ki67 expression in CLL cells in both the PB and spleen of ibrutinib treated mice (mean reduction >50%, Figure 6c-e; *P*≤.006). Thus ibrutinib potently and selectively inhibits tumor proliferation *in vivo*.

## Discussion

Inhibitors of BCR signaling have achieved impressive responses in early clinical trials.^25-28^ Although much progress has been made in understanding the biologic effects of these agents *in vitro*, many questions remain to be investigated. Given the importance of tumor host interactions in the lymph node and the difficulty of accessing nodal disease in patients, the development and use of *in vivo* model systems is an important avenue of research. Herein, we confirm that the recently described CLL xenograft model in NSG mice supports tumor proliferation at a similar rate as seen in patients.^39^ In addition, we have expanded the characterization of the model to demonstrate that CLL activation in the murine spleen involves activation of BCR and NF-κB signaling in a similar manner as we have previously described in the human LN.^3^ Notably, this conclusion is based on direct comparisons of matched tumor samples of the same patients donating the PBMCs used for xenografting. For consistency between experiments we used viably frozen samples for all xenografting experiments. Given the high viability of the samples and the fact that the xenografted cells undergo activation and proliferation in the murine host, we expect that results using fresh cells would yield comparable results. Thus, our studies support the use of this model to further investigate the role of tumor-microenvironment interactions in the pathogenesis of CLL and as a preclinical tool to evaluate targeted interventions.

Recently, adoptively transferred cells from the TCL1 transgenic model were used to study the anti-tumor effects of fostamatinib and ibrutinib *in vivo*.^33, 45^ Here, we extend these studies by showing that ibrutinib disrupted BCR and NF-κB signaling pathways in xenografted CLL cells in the spleen microenvironment *in vivo* and selectively reduced proliferation of the tumor cells but not of T-cells. In addition, ibrutinib decreased the viability of xenografted CLL cells in the mouse spleen. This data corroborates previous work by Herman et al. and Ponader et al. demonstrating that ibrutinib can inhibit activation, proliferation and survival of CLL cells *in vitro* at clinically relevant doses.^31, 39^ Combined, these effects likely contribute to the decrease in tumor burden in the spleens of ibrutinib treated mice. Likewise, CLL patients treated with ibrutinib often experience a rapid decrease in disease burden in tissue sites, while the absolute lymphocyte count in the PB increases.^15, 26, 28^ Mirroring these observations in patients, ibrutinib resulted in an increase in CLL numbers in the PB concurrently with a decreased tumor burden in the spleen. In summary, inhibition of BTK was sufficient to substantially inhibit the supportive effect of the microenvironment on tumor proliferation and survival *in vivo*, most likely through inhibition of BCR and NF-κB signaling.

Activation of BCR signaling in human CLL cells in the mouse microenvironment might surprise at first. However, because unselected human PBMCs are injected, there is a significant transfer of T-cells, and both T- and CLL cells co-localize in follicular structures in the mouse spleen. Thus, the CLL cells are embedded in a partially humanized environment. As reported by others, T-cells are required for CLL proliferation and contribute to the dynamic upregulation of CD38 on xenografted tumor cells.^39, 41^ In the present study we did not distinguish distinct T-cell subsets; however data by Bagnara et al. suggests that expansion of the CD4+ subset is indispensible for CLL proliferation and survival.^39^ Activated T-cells secrete cytokines, can provide co-stimulatory signals for BCR activation, and express CD40 ligand, which may contribute to NF-κB activation in xenografted CLL cells.^1^ In this environment there is likely also significant cellular apoptosis with upregulation of antigens on apoptotic cells that have been shown to stimulate the BCR of CLL cells.^21, 46^ Thus, the spleen microenvironment in these xenografted mice may recapitulate many of the complexities of the human microenvironment. Furthermore, a recent study identified a CLL cell autonomous BCR signal that is generated from the engagement of the antigen binding site of one BCR by the framework region of another.^47^ Thus, it is possible that in a conducive microenvironment the BCR on CLL cells is activated in an autonomous fashion.^48^

Because CLL cells from different patients can be studied in the NSG xenotransplant model, it may be amenable to recapitulate the heterogeneity of CLL. In addition observations in the murine model can be directly correlated to disease characteristics in the patient donating the cells for xenografting; as we have done here in regards to tumor cell activation and proliferation. Our analysis was based on cells from 10 different patients that span the spectrum of CLL patients who eventually require treatment, thus a majority are of the IGHV unmutated type as is typically seen in treatment studies. It will be important to broaden the use of the model to investigate tumor behavior in respect to distinct disease characteristics such IGHV mutational status or distinct genetic lesions. In addition, it will be of interest to test whether primary tumor samples from patients with variable clinical responses to kinase inhibitors will also show differential responses in the xenograft model. In this regard, the model may be of particular value to study mechanism of action and resistance to the emerging targeted agents in CLL.

## Supplementary Material

1

2

## Figures and Tables

**Table 1:** Patient characteristics

Patient ID	Sex/Age (years)	Rai Stage	IGHV gene, % germline	CD38^1^	CD49d^1^	FISH	LN proliferation centers^2^	TTT, mo	Growth Rate, %/day^3^	Symbol
CLL_01	M/58	3	3-30, 100%	+	+	del 6q	prominent	9.6	0.91	**△**
CLL_02	F/63	2	3-21, 100%	+	+	normal	typical	21.6	0.68	**×**
CLL_03	M/50	4	3-30, 100%	-	-	del 11q	typical	30	0.93	**#**
CLL_04	M/53	2	1-69, 100%	+	-	del 13q/11q	typical	10	0.26	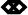
CLL_05	M/68	3	3-49, 99.3%	+	-	del 13q/11q	atypical	49	ND	**+**
CLL_06	M/58	2	1-02, 100%	+	+	normal	NA	47	ND	*
CLL_07	M/53	4	3-30, 99.7%	+	+	del 13q	NA	120	0.85	**□**
CLL_08	F/77	3	4-b, 95.2%	+	-	del 13q	typical	305	0.31	**▽**
CLL_09	F/60	2	3-21, 96.3%	+	-	del 13q/11q	typical	79.7	0.29	**○**
CLL_10	F/48	3	4-34 92.4%	-	-	del 13q	atypical	48	ND	**◊**

1 By flow cytometry, >30% of cells staining for CD38 or CD49d, respectively were considered positive.

2 The pattern of proliferation centers was assessed on lymph node biopsies where available and scored as typical (small areas with increased frequency of larger cells with more open chromatin that are widely spaced), prominent (larger, more frequent proliferation centers) and atypical (confluent proliferation centers).

3 Measured by flow cytometry as the fraction of cells showing diluting out of CFSE between two timepoints, divided by the number of days

NA = not available; ND = not determined
