# Supplementary material for: Modeling Tumor-Host Interactions of Chronic Lymphocytic Leukemia in Xenografted Mice to Study Tumor Biology and Evaluate Targeted Therapy
Source: Leukemia. Author manuscript; Available in PMC 2014 Aug 8. (PMC4126654; doi:10.1038/leu.2013.131)

Supplementary Figure S1

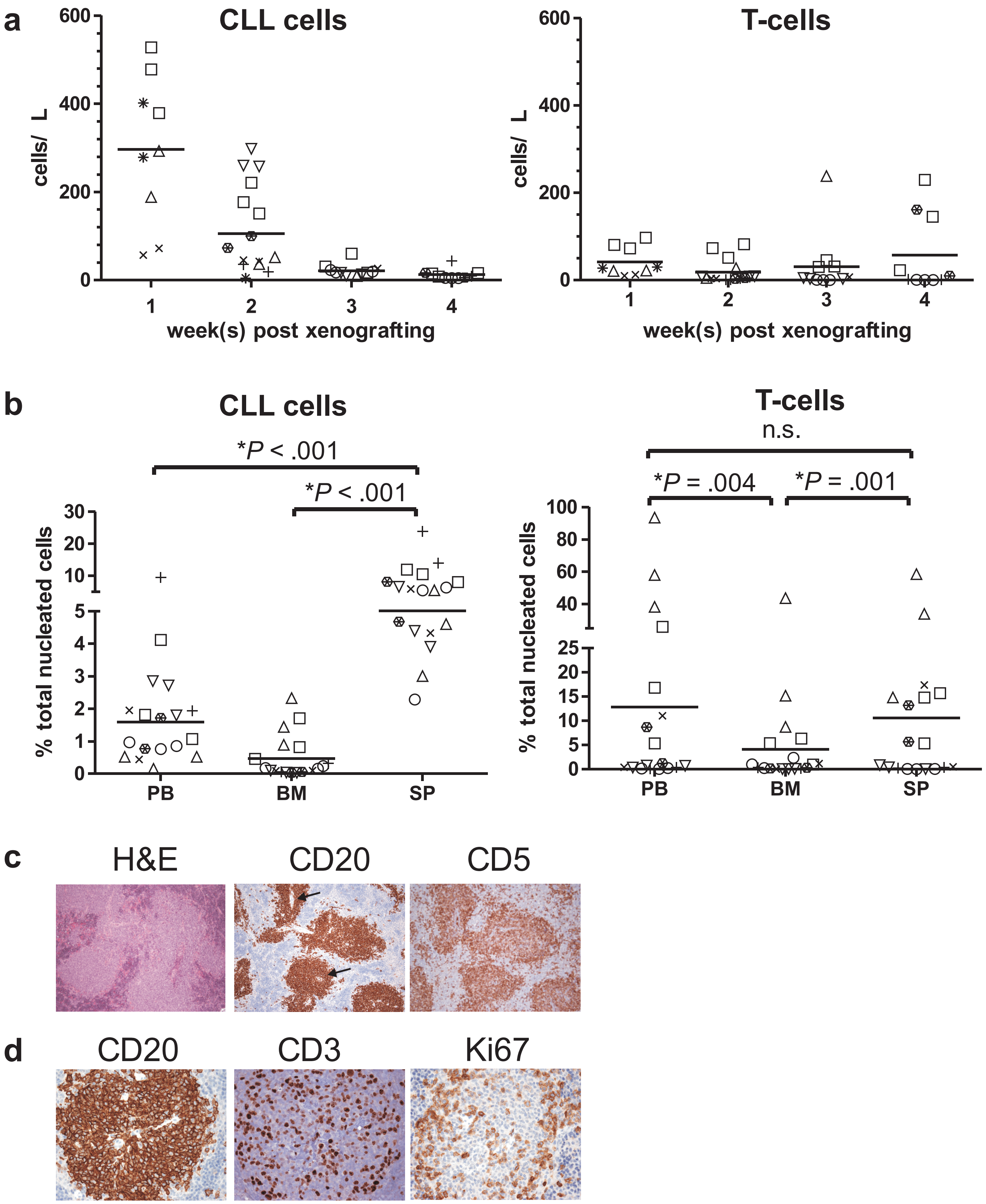

Supplementary Figure S2

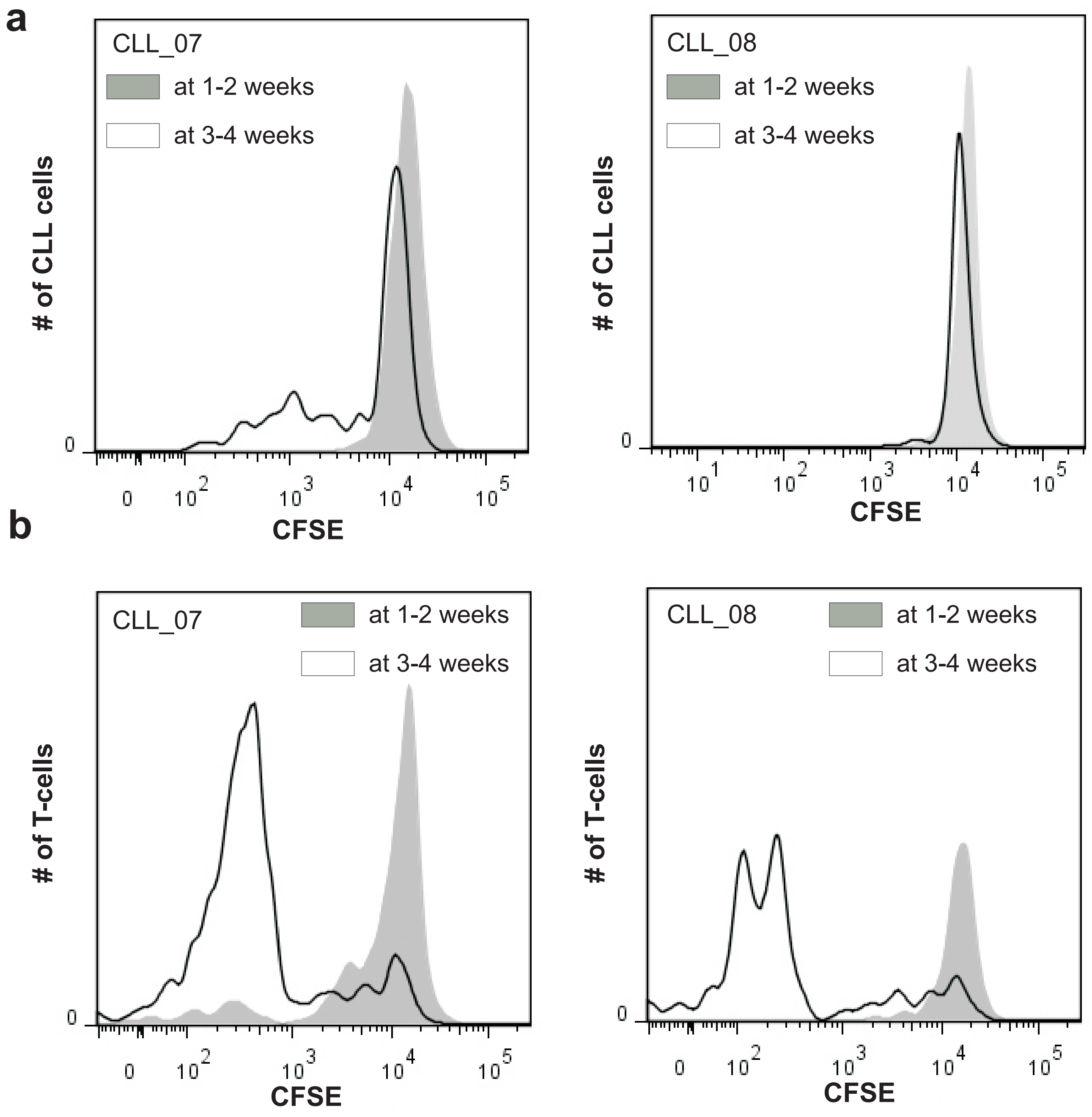

Supplementary Figure S3

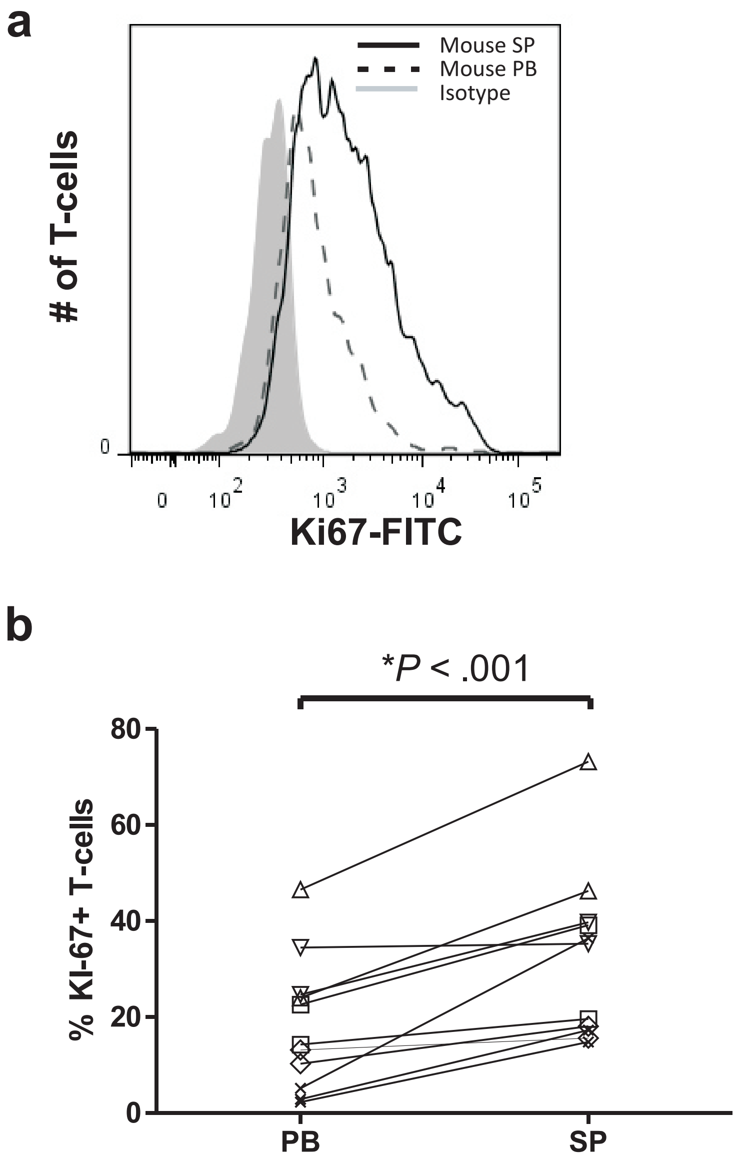

Supplementary Figure S4

a

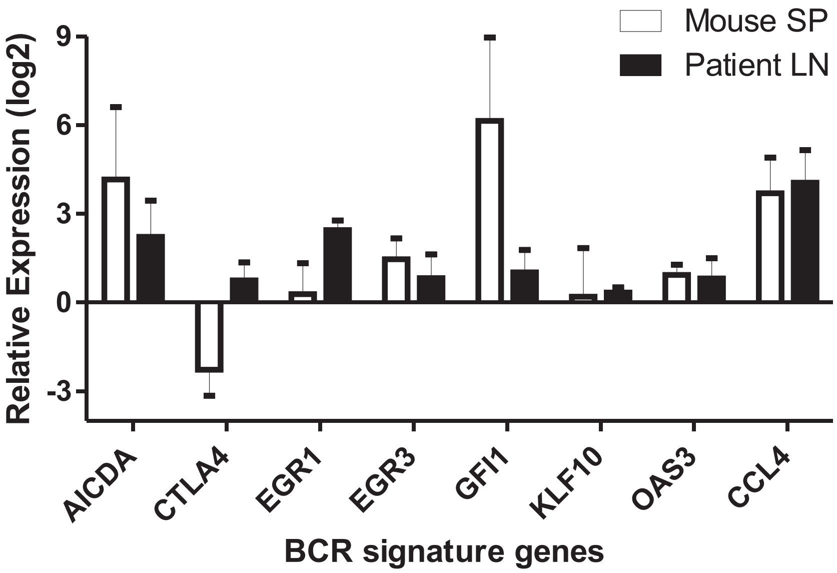

b

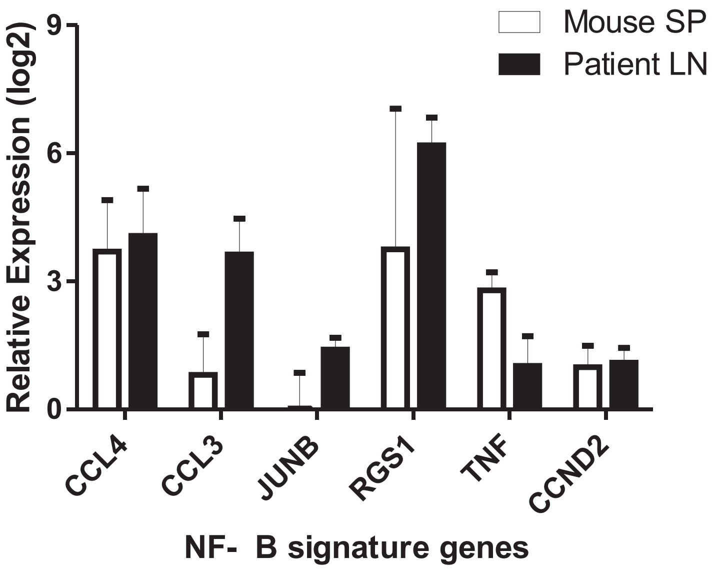

Supplementary Figure S5

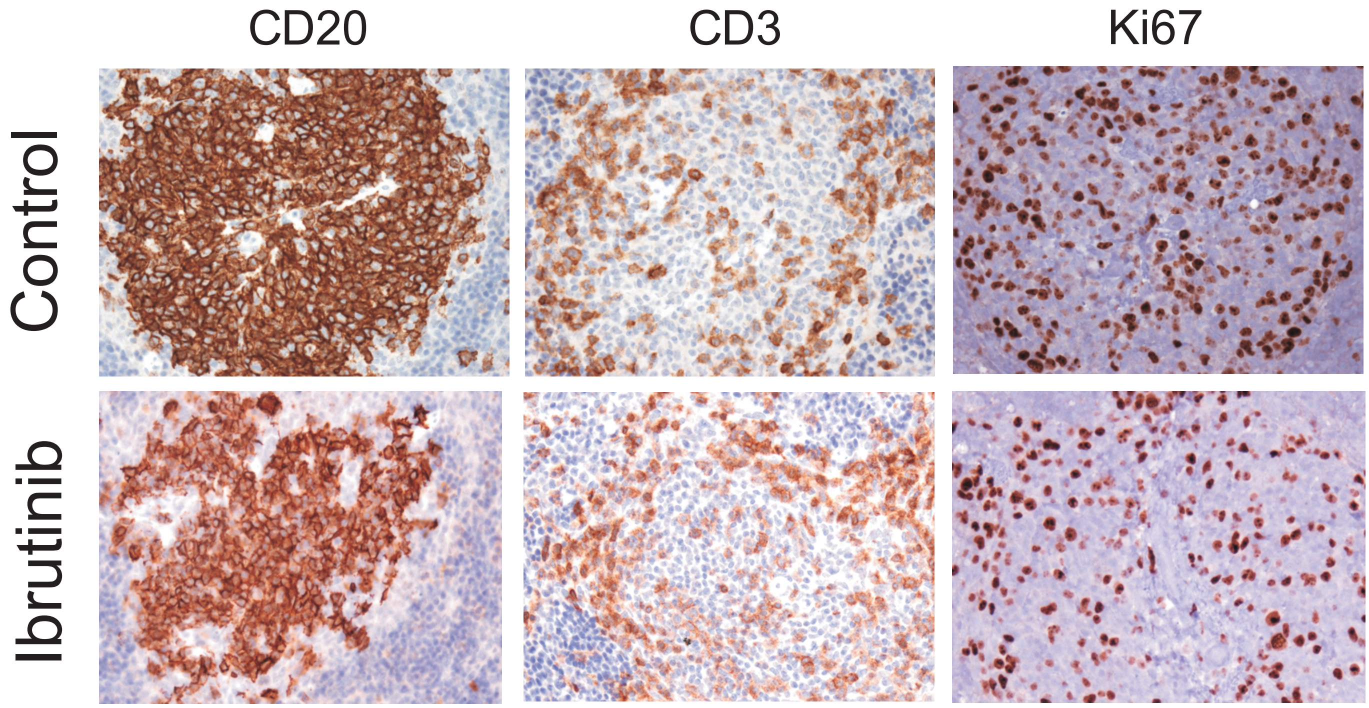

Supplementary Figure S6

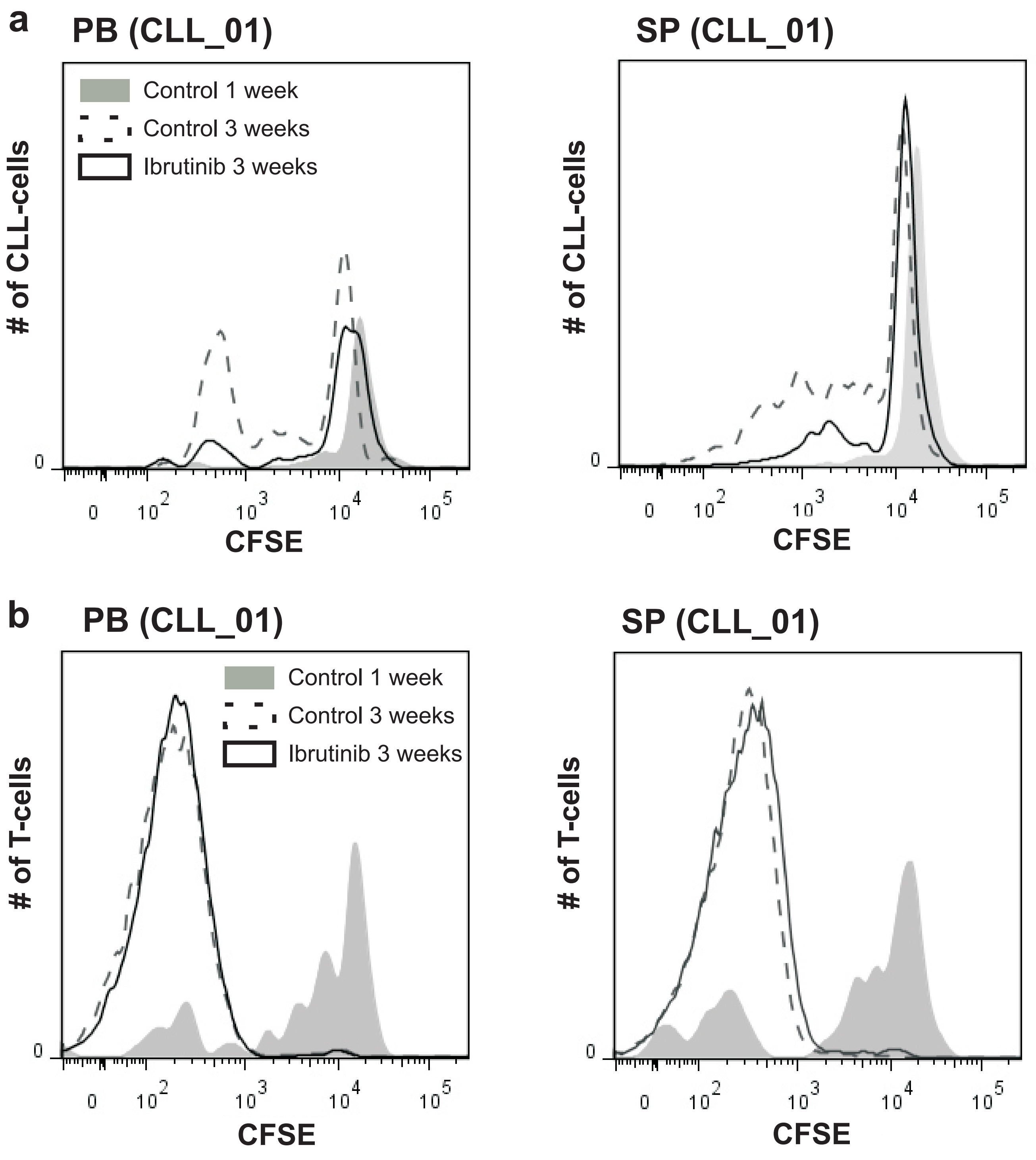

Supplementary Figure S7

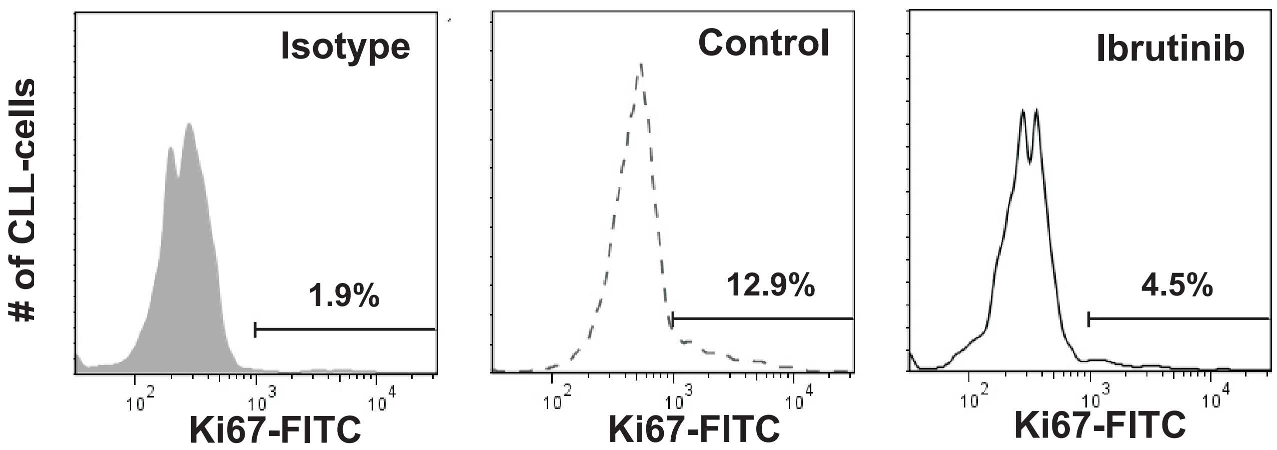

Supplement: 1 [file NIHMS549954-supplement-1.pdf]
