# Supplementary material for: Modeling Tumor-Host Interactions of Chronic Lymphocytic Leukemia in Xenografted Mice to Study Tumor Biology and Evaluate Targeted Therapy
Source: Leukemia. Author manuscript; Available in PMC 2014 Aug 8. (PMC4126654; doi:10.1038/leu.2013.131)
Supplement: 2 [file NIHMS549954-supplement-2.docx]

**Supplementary Table S1.** Assay IDs for Genes analyzed by RT-PCR

| **Gene symbol** | **Gene Name** | **Assay ID** | **Gene Signature^1^** |
| --- | --- | --- | --- |
| *CCL3* | chemokine (C-C motif) ligand 3 | Hs00234142_m1 | NF-κB |
| *CCL4* | chemokine (C-C motif) ligand 4 | Hs99999148_m1 | BCR, NF-κB |
| *EGR1* | early growth response 1 | Hs00152928_m1 | BCR |
| *OAS3* | 2-5-oligoadenylate synthetase 3, 100kDa | Hs00196324_m1 | BCR |
| *18S* | Eukaryotic 18S rRNA | Hs99999901_s1 | Control |
| *TNF* | tumor necrosis factor | Hs99999043_m1 | NF-κB |
| *PCNA* | proliferating cell nuclear antigen | Hs00952870_g1 | Proliferation |
| *EGR3* | early growth response 3 | Hs00231780_m1 | BCR |
| *B2M* | beta-2-microglobulin | Hs00187842_m1 | Control |
| *CDT1* | chromatin licensing and DNA replication factor 1 | Hs00417193_g1 | Proliferation |
| *CCND2* | cyclin D2 | Hs00153380_m1 | NF-κB |
| *GFI1* | growth factor independent 1 transcription repressor | Hs00193366_m1 | BCR |
| *KLF10* | Kruppel-like factor 10 | Hs00921811_m1 | BCR |
| *RGS1* | regulator of G-protein signaling 1 | Hs00175260_m1 | NF-κB |
| *CTLA4* | cytotoxic T-lymphocyte-associated protein 4 | Hs00175480_m1 | BCR |
| *AICDA* | activation-induced cytidine deaminase | Hs00757808_m1 | BCR |
| *VCP* | valosin-containing protein | Hs00997642_m1 | Control |
| *RRM2* | ribonucleotide reductase M2 | Hs01072069_g1 | Proliferation |
| *JUNB* | jun B proto-oncogene | Hs00357891_s1 | NF-κB |

**^1^**As described in Ref (3).

**Supplementary Figure S1.** Blood and tissue localization of xenografted human PBMCs. (**a**) Flow cytometric enumeration of absolute cell count of CLL cells (CD45+/CD19+/CD5+; left panel) and T-cells (CD45^+^, CD19^−^, CD5^+^; right panel) in the blood of xenografted mice over 4 weeks from the injection of 1x10^8^ PBMCs. Each symbol represents a unique patient (listed in Table 1), each data point represents one mouse. (**b**) CLL cells preferentially localize to the spleen of NSG mice. Shown is the percentage of CLL cells and T-cells among the total number of nucleated cells (defined by FSC and SSC properties) in peripheral blood (PB), bone marrow (BM), and spleen (SP) in mice sacrificed 3-4 weeks after xenografting. Both CLL cells and T- cells are only a minor fraction of the total number of nucleated cells in the BM. Student’s t-test was used to test for significance. (**c**) Sections of murine spleens obtained at 3-4 weeks post xenografting were stained with Hematoxylin and Eosin (H&E), and for CD5, CD20. Images of a representative sample were captured at 100X magnification on an Olympus Bx41 microscope. There is a nodular pattern of infiltration by CLL cells, typically surrounding blood vessels (arrows). **d**) Immunohistochemical stains for CD20, and Ki67; captured at 400X magnification on an Olympus Bx41 microscope.

**Supplementary Figure S2.** Proliferation of human cells in the NSG mice. (**a-b**) PBMCs from CLL patients were labeled with 0.5 µM CFSE before injection. Histograms depict the step-wise dilution of CFSE in xenografted human cells that have undergone cell division from two representative experiments with cells from two different patients. PB samples were analyzed at 1-2 weeks from xenografting (gray filled area) and again 2 weeks later (solid lines). The rate of CLL cell proliferation (**a**) differs between patients, and T-cells proliferate (**b**) faster than CLL cells.

**Supplementary Figure S3.** T-cell proliferation in the peripheral blood and spleen. (**a**) A representative histogram demonstrating increased frequency of Ki67 positive T-cells in mouse spleen (SP) compared to the matched mouse PB 3-4 weeks post xenografting is shown. (**b**) Summary of experiments from six different patients (identified by symbols, Table 1) with 2-3 mice per patient analyzed (n=13 total). Student’s paired t-test was used to test for significance.

**Supplementary Figure S4.** CLL cells in secondary lymphoid tissues upregulate BCR and NF-κB target genes. BCR and NF-κB target genes (as described in Ref (3)) were measured using quantitative PCR in mRNA from purified CLL cells. Shown is the mean (± SEM) expression for each gene in CLL cells from the indicated tissue site divided by its expression in the corresponding PB cells as described in Materials and Methods. CLL cells were CD19+ purified from the spleens of xenografted mice (2-4 spleens per patient) and from the corresponding patients’ LN and PB (n=3).[^3^](#_ENREF_3)

**Supplementary Figure S5:** Proliferation in lymphoid aggregates in the mouse spleen is reduced by ibrutinib. Immunohistochemical staining of spleen tissue from control vs. ibrutinib treated mice suggests a decrease in CD20+ cells as well as decreased in KI67+ cells in the lymphoid nodules of ibrutinib treated mice. Images of murine spleens obtained at 3-4 weeks from xenografting stained for CD3, CD20, and Ki67 were captured at 400-fold magnification on an Olympus Bx41 microscope (Center Valley, PA).

**Supplementary Figure S6.** CLL cell proliferation is reduced in ibrutinib treated mice compared to controls (**a-b**) A representative histogram depicts CFSE staining in human CLL (**a**) or T-cells (**b**) isolated from PB or spleen (SP) as indicated. The gray filled area shows CFSE staining in a control mouse 1 week after xenografting and identifies cells not having undergone cell division (those with maximum intensity of CFSE staining). Two weeks later, a substantial fraction of CLL cells in the control mouse has undergone cell division, but much less so in the ibrutinib treated mouse (shown in **a**). T-cell proliferation is not affected by ibrutinib (**b**).

**Supplementary Figure S7.** Ibrutinib reduces the percentage of CLL cells positive for Ki67. Panels show the measurement of % Ki67 positive CLL cells in spleens at sacrifice for isotype (left panel), control mouse (middle panel) and treated mouse (right panel).
